# Renal Salvage in Pyonephrosis: A Retrospective Observational Study at a Tertiary Care Center in North India

**DOI:** 10.7759/cureus.102343

**Published:** 2026-01-26

**Authors:** Syed Shakeeb Arsalan, Aamir B Raina, Abdul R Khawaja, Arif Hamid, Sajad A Malik, Sajad A Para, Saqib Mehdi, Syed Aadil S Andrabi, Gokul Kannan, Uvaisullah Quadir

**Affiliations:** 1 Department of Urology, Sher-i-Kashmir Institute of Medical Sciences, Srinagar, IND

**Keywords:** double-j stent, nephrectomy, percutaneous nephrostomy, pyonephrosis, urolithiasis

## Abstract

Introduction

Pyonephrosis is a urological emergency associated with a high risk of sepsis and irreversible renal damage. Early diagnosis and timely drainage are critical for renal salvage and for reducing morbidity and mortality. Data from tertiary care centers in our region are limited. This study evaluates the clinical profile, management, and outcomes of pyonephrosis over a five-year period at a tertiary referral center.

Materials and methods

This retrospective study included 90 adult patients diagnosed with pyonephrosis at the Department of Urology, Sher-i-Kashmir Institute of Medical Sciences (SKIMS), Srinagar, between August 2020 and July 2025. The objectives of the study were to evaluate renal salvage outcomes in pyonephrosis and to identify clinical, radiological, and etiological factors influencing salvageability. Demographic, clinical, laboratory, imaging, and treatment details were analyzed. Statistical analysis was performed using IBM SPSS Statistics version 25.

Results

The mean age was 45.1 years, with male predominance (57%); 83% of patients were from rural areas. Urolithiasis was the most common etiology (46.7%), followed by pelvi-ureteric junction (PUJ) obstruction (16.7%) and malignancy (13.3%). Left-sided involvement was seen in 47%, right-sided in 43%, and bilateral disease in 10%. Initial urinary drainage was achieved by percutaneous nephrostomy (PCN) in 50%, Double-J (DJ) stenting in 16.6%, and combined DJ stenting with PCN in 20%. Definitive treatment included nephrectomy in 46.7%, percutaneous nephrolithotomy (PCNL) in 24.1%, and pyeloplasty in 10.3%. Renal salvage was achieved in 53.3% of patients. Factors significantly associated with non-salvage included older age (p = 0.046), acute kidney injury (p < 0.0001), sepsis at presentation (p < 0.0001), parenchymal thickness < 5 mm (p < 0.0001), prolonged symptom duration (p < 0.0001), and severe hydronephrosis (p < 0.0001).

Conclusions

Urolithiasis remains the leading cause of pyonephrosis. Prompt imaging and early urinary decompression are essential for renal salvage and improved clinical outcomes.

## Introduction

Pyonephrosis is a urological emergency characterized by suppurative infection of an obstructed renal collecting system, leading to progressive renal damage and a high risk of systemic sepsis. It represents an advanced stage of infected hydronephrosis, although the clinical distinction between the two entities is often challenging. Obstruction, most commonly due to calculus disease, results in urinary stasis, increased intrarenal pressure, secondary infection, renal ischemia, and eventual parenchymal necrosis [1]. Clinically, patients often present with flank pain, fever with rigors, and varying degrees of hemodynamic instability. Delayed diagnosis and drainage are associated with significant morbidity, irreversible loss of renal function, and reported mortality rates of up to 20% [2]. Pyonephrosis has been reported to complicate approximately 5-12% of cases of hydronephrosis or severe pyelonephritis [3].

Urolithiasis is the most common etiological factor worldwide, accounting for 60-83% of cases [4]. Stones typically lodge at anatomical narrowings such as the pelvi-ureteric or ureterovesical junctions, leading to obstruction and secondary infection. Other contributing factors include pelvi-ureteric junction (PUJ) obstruction, ureteric strictures, renal cystic disease, and malignancy. In endemic regions, infective ureteric strictures due to tuberculosis or schistosomiasis contribute to an additional 3-7% of cases [5].

Gram-negative enteric organisms predominate, with *Escherichia coli* accounting for 30-40% of isolates, followed by *Klebsiella pneumoniae* (15-20%) and *Pseudomonas aeruginosa* (5-10%) [6]. Gram-positive organisms such as *Staphylococcus aureus* and *Enterococcus *species are less frequent but may be encountered in immunocompromised patients or those with indwelling urinary devices [7].

Ultrasonography is typically the initial imaging modality, demonstrating internal echoes or debris within a dilated collecting system in over 80% of cases [8]. However, its ability to differentiate infected from sterile hydronephrosis is limited. Contrast-enhanced computed tomography (CECT) of the abdomen and pelvis has therefore emerged as the imaging modality of choice, with characteristic findings including pelvic wall thickening (>2 mm), perinephric fat stranding, renal parenchymal hypoenhancement, and fluid-debris levels [9].

Urgent decompression of the obstructed system remains the cornerstone of management. Percutaneous nephrostomy (PCN) and retrograde double-J (DJ) stenting are the two most commonly employed techniques. PCN provides rapid and effective drainage, with reported success rates exceeding 90%, and has been shown to result in superior clinical improvement compared to DJ stenting in several studies [7,10]. Although less invasive, DJ stenting may be less effective in the presence of thick purulent debris, with secondary conversion to PCN required in 20-30% of cases [11].

Comorbidities such as diabetes mellitus and chronic kidney disease (CKD) significantly influence disease severity and outcomes. Diabetes predisposes patients to severe infections due to impaired host immunity, while pre-existing renal dysfunction is associated with poorer renal recovery and higher complication rates [12].

Despite established management principles, limited data exist from tertiary care centres in the Indian subcontinent evaluating predictors of renal salvage in pyonephrosis [13]. As a regional referral centre, Sher-i-Kashmir Institute of Medical Sciences (SKIMS), Soura, manages a substantial number of pyonephrosis cases using standardized diagnostic and therapeutic protocols. This retrospective study aims to evaluate the demographic profile, clinical presentation, imaging findings, microbiological spectrum, management strategies, and outcomes of patients with pyonephrosis treated at our institution, thereby contributing to existing regional evidence and informing optimal management practices.

## Materials and methods

This retrospective observational study was conducted in the Department of Urology, SKIMS, Srinagar, a tertiary referral center, over a five-year period from August 2020 to July 2025. All adult patients (≥18 years) admitted and managed for pyonephrosis during the study period were included. Pyonephrosis was diagnosed based on a combination of clinical features, laboratory parameters, radiological findings, and confirmation at urinary drainage (PCN or DJ stenting) or surgical intervention (nephrectomy). Patients with incomplete medical records and those initially managed at other institutions were excluded. A convenience sampling method was used, including all eligible cases identified from institutional medical records.

The primary objective of this study was to evaluate renal salvage outcomes in patients presenting with pyonephrosis at a tertiary care centre. The secondary objectives were to analyze the etiological factors, clinical presentation, microbiological profile, management strategies, and to identify clinical and radiological predictors associated with renal salvage or the need for nephrectomy.

Data were retrospectively extracted from the hospital medical records department and urology and radiology databases using a predesigned structured proforma. Collected variables included demographic characteristics (age, sex, residence, smoking status); clinical features (presenting symptoms, duration of symptoms, and comorbidities such as diabetes mellitus, chronic kidney disease, hypertension, and immunosuppression); laboratory parameters (complete blood count, serum creatinine, blood urea, serum electrolytes, CRP, urine microscopy and culture, pus culture, and blood culture where available); radiological findings (USG and CECT abdomen and pelvis); and microbiological profile (organisms isolated from urine or aspirated pus samples and antimicrobial susceptibility patterns). Management-related variables included the timing and mode of initial drainage (PCN, DJ stenting, or combined procedures), indications and timing of nephrectomy, and perioperative complications graded according to the Clavien-Dindo classification. Outcome measures assessed were length of hospital stay, complication rates, renal salvage, nephrectomy rates, and in-hospital mortality.

The study was approved by the Institutional Ethics Committee of SKIMS. In view of the retrospective study design, the requirement for informed consent was waived. Patient confidentiality was maintained by anonymizing all identifiable information during data collection and analysis.

Statistical analysis

Data were entered and analyzed using IBM SPSS Statistics for Windows, Version 25.0 (IBM Corp., Armonk, New York, USA). Continuous variables are presented as mean ± SD, and categorical variables are expressed as frequencies and percentages. Comparisons between the renal salvage and non-salvage (nephrectomy) groups were performed using the independent Student’s t-test for continuous variables. Categorical variables were compared using the Chi-square test, and Fisher’s exact test was applied when expected cell counts were less than five. All statistical tests were two-tailed, and a p value < 0.05 was considered statistically significant.

## Results

A total of 90 patients were included in the study. The mean age at presentation was 45.1±11.9 years (range: 18-77 years), with a male predominance, with 51 (56.7%) males and a male-to-female ratio of 1.3:1. Most patients were from rural areas, i.e., 75 (83%), while 15 (17%) patients resided in urban settings. Left-sided renal involvement was observed in 42 patients (47%), right-sided in 39 (43%), and bilateral disease in 9 patients (10%). Stone disease was the most common cause of pyonephrosis, accounting for 42 patients (46.7%). Other etiologies included PUJ obstruction in 15 patients (16.7%), pelvic malignancy in 12 (13.3%), genitourinary tuberculosis in 7 (7.8%), obstructive megaureter in 6 (6.7%), benign ureteric strictures in 5 (5.6%), and xanthogranulomatous pyelonephritis in 3 patients (3.3%) (Table 1).

**Table 1:** Demographic characteristics and etiology. PUJ: Pelviureteric Junction; GUTB: Genitourinary Tuberculosis.

Variable	Category	Number	Percentage
Age (years)	Mean ± SD	45.1 ± 11.9	-
Sex	Male	51	56.70%
	Female	39	43.30%
Residence	Rural	75	83.30%
	Urban	15	16.70%
Laterality	Bilateral involvement	9	10.00%
	Right side	39	43.30%
	Left side	42	46.70%
Etiology	Stone disease	42	46.70%
	PUJ obstruction	15	16.70%
	GUTB	7	7.80%
	Xanthogranulomatous pyelonephritis	3	3.30%
	Benign ureteric strictures	5	5.60%
	Pelvic malignancy	12	13.30%
	Obstructive megaureter	6	6.70%

The most common presenting symptom was flank pain in 76.6% of patients (n = 69), followed by fever in 70% (n = 63). Other symptoms included vomiting in 13.3% (n = 12), dysuria in 8.8% (n = 8), hematuria in 6.6% (n = 6), and oliguria in 6.6% (n = 6). Less common presentations included storage lower urinary tract symptoms in 3.3% (n = 3), an abdominal lump in 2.2% (n = 2), and discharge through the skin in 1.1% (n = 1). Among the 90 patients, 22 (23.3%) were smokers.

Comorbid conditions were absent in 54 patients (60%). Among those with comorbidities, hypertension was the most frequent (n = 9), followed by combined CKD with hypertension (n = 9), diabetes mellitus (n = 6), hypothyroidism (n = 6), diabetes mellitus with hypertension (n = 3), and isolated CKD (n = 3).

Percutaneous nephrostomy was the most common initial intervention, performed in 45 patients (50%). DJ stenting, either alone or in combination with PCN, was performed in 33 patients, while PCN with additional percutaneous drainage (PCD) was required in 9 patients. Three patients did not undergo any initial drainage procedure. Nephrectomy was the most frequent definitive treatment, performed in 42 patients (46.7%), followed by percutaneous nephrolithotomy (PCNL) in 21 (24%) and pyeloplasty in 9 (10%). Other interventions included segmental ureteric resection with ureteric reimplantation, renal transplantation, and referral for oncological management after drainage (Table 2).

**Table 2:** Initial decompression procedures and definitive treatment modalities. PCN: Percutaneous nephrostomy; DJ: Double-J stent; PCD: Percutaneous drainage; PCNL: Percutaneous nephrolithotomy.

Treatment phase	Procedure	Number	Percentage
Initial decompression	PCN	45	50.00%
	DJ stenting	15	16.70%
	DJ stenting + PCN	18	20.00%
	PCN + PCD	9	10.00%
	No initial intervention	3	3.30%
Definitive treatment	Nephrectomy	42	46.70%
	PCNL	21	24.10%
	Pyeloplasty	9	10.30%
	Ureteric reimplantation	6	6.90%
	Renal transplantation (post-nephrectomy)	3	3.30%
	Referral to medical oncology after drainage	12	13.30%

**Figure 1:**
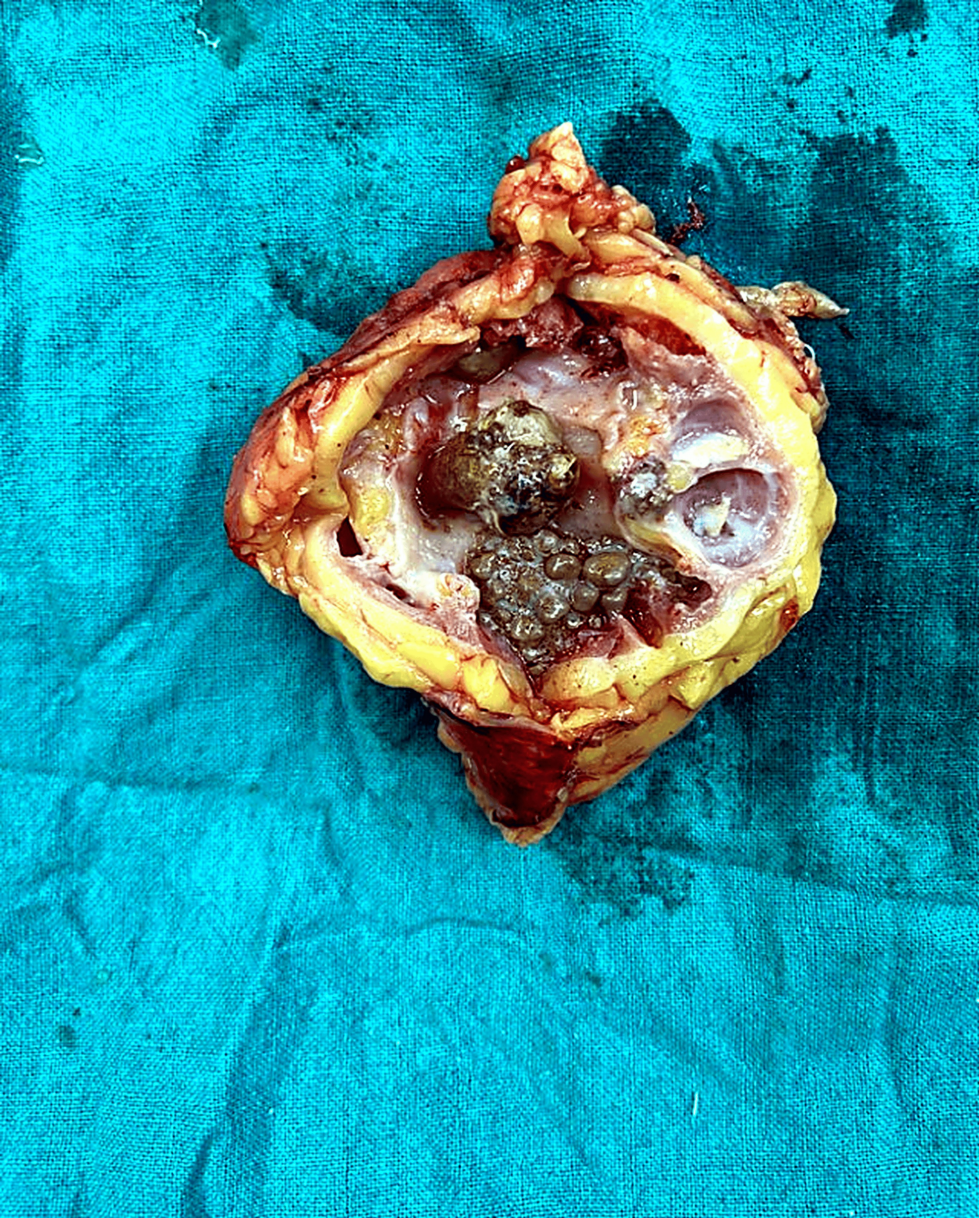
Nephrectomy specimen of a pyonephrotic kidney showing parenchymal destruction and multiple stones.

Among patients undergoing nephrectomy, 23 procedures (54.8%) were performed using an open approach and 19 (45.2%) laparoscopically (Figure 1).

Pus cultures obtained following PCN in 72 patients showed microbial growth in all cases. *Escherichia coli* was the most common isolate in 42 cases (58.3%), followed by *Pseudomonas* species in 10 (14.3%). Acid-fast bacilli positivity was observed in 10 cases (14.3%). *Klebsiella* species and *Staphylococcus aureus* were each isolated in 5 (7.1%) patients.

At presentation, sepsis was the most common complication, occurring in 27 (30%) patients, while 27 patients (30%) had no complications. Other complications included perinephric and parietal wall collections, renal or psoas abscesses, and urinary fistulae. Following nephrectomy, 33 patients had an uneventful postoperative course, while surgical site infection was noted in nine patients. Isolated complications included postoperative fever with sepsis, septic shock, pleural effusion with lung consolidation, and prolonged ileus. Among patients undergoing pyeloplasty, urine leak was observed in three cases.

Overall, renal salvage was achieved in 48 patients (53.3%), while nephrectomy or non-salvageable kidneys accounted for 42 cases (46.7%). Renal salvage was significantly associated with younger age (42.2 ± 11.4 vs. 48.3 ± 12.2 years; p = 0.017). Acute kidney injury and sepsis at presentation were more frequent in the non-salvage group (p < 0.0001 for both). Parenchymal thickness >8 mm was strongly associated with renal salvage, whereas thickness <5 mm was predominantly observed in non-salvageable kidneys (p < 0.0001). Mean duration of symptoms was 15 ± 5 days in the salvageable kidney group and 28 ± 5 days in the non-salvageable group. Patients with shorter symptom duration had higher salvage rates (p < 0.0001). Mild-to-moderate hydronephrosis was associated with renal salvage, while severe hydronephrosis correlated with non-salvage (p < 0.0001). Etiology also influenced outcomes, with stone disease showing a significant association with renal salvage (p = 0.0007), whereas obstructive megaureter was associated with poor salvage (p = 0.012); other etiologies did not demonstrate statistically significant differences (Table 3).

**Table 3:** Predictors of renal salvage in patients with pyonephrosis. AKI: Acute kidney injury; PUJ: Pelviureteric junction. Continuous variables were compared between salvaged and non-salvaged groups using the independent Student’s t-test. Categorical variables were compared using the Chi-square test, and Fisher’s exact test was applied when appropriate. All p-values shown in the table were calculated using these tests. A p-value < 0.05 was considered statistically significant.

Predictor	Salvaged kidney (n)	Non-salvaged (nephrectomy) (n)	p-value
Age (years), mean ± SD	42.2 ± 11.4	48.3 ± 12.2	0.017
AKI at presentation	9	33	<0.0001
Sepsis at presentation	11	29	<0.0001
Parenchymal thickness			<0.0001
- <5 mm	1	35	
- 5-8 mm	12	7	
- >8 mm	35	0	
Duration of symptoms (days), mean ± SD	15 ± 5	28 ± 5	<0.0001
Grade of hydronephrosis			<0.0001
- Mild	18	0	
- Moderate	24	2	
- Severe	6	40	
Etiology			
- Stone disease	30	12	0.0007
- PUJ obstruction	6	9	0.2
- Genitourinary tuberculosis	3	4	0.7
- Xanthogranulomatous pyelonephritis	0	3	0.086
- Ureteric stricture	2	3	0.66
- Malignancy	7	5	1
- Obstructive megaureter	0	6	0.012

## Discussion

Pyonephrosis represents an advanced stage of infected obstructive uropathy and is associated with a high risk of sepsis, irreversible renal damage, and mortality if not managed promptly. Urgent recognition and decompression are central to management; however, renal recovery remains variable and depends on the underlying etiology, duration of obstruction, extent of parenchymal damage, and severity of systemic involvement. The present study provides contemporary data from a high-volume tertiary referral center in North India and contributes to the limited regional literature describing the clinical spectrum, management strategies, and renal salvage outcomes in pyonephrosis.

Urolithiasis was the predominant etiology in our cohort, consistent with reports from India, China, and other Asian regions where stone disease accounts for the majority of cases. Several authors have reported calculus disease as the cause in 70-90% of pyonephrosis cases. Hemal AK et al. documented stones in 82% of patients, while Liu J et al. reported calculus disease in more than 75% of cases [14,15]. Similarly, Singh et al. from North India observed stones in 88% of patients with pyonephrosis. The demographic profile in our study, with a mean age in the mid-40s and a slight male predominance, mirrors patterns seen in Indian series and likely reflects regional stone epidemiology and delayed healthcare access. The predominance of patients from rural areas is consistent with findings by Nerli RB et al. and others, who emphasized delayed presentation, higher stone burden, and advanced infective complications in rural populations [16].

Clinically, fever, flank pain, and systemic toxicity were the most common presenting features, with a significant proportion of patients exhibiting sepsis and AKI. The strong association between AKI and non-salvageability observed in our study is supported by experimental models of obstructive nephropathy described by Klahr S et al., demonstrating impaired renal perfusion, tubular injury, and irreversible nephron loss with prolonged obstruction [17]. USG was used as the initial imaging modality, while CECT, as demonstrated by Stojadinović M et al., Tamburrini S et al., and Florido C et al., provides additional diagnostic information by differentiating infected from sterile hydronephrosis and aids in planning the most appropriate drainage approach [8-10].

Microbiological analysis revealed a predominance of Gram-negative organisms, particularly *Escherichia coli*, *Klebsiella*, and *Pseudomonas*, consistent with findings reported by St Lezin M et al. and Rabii R et al. [18,19]. This distribution reflects the typical pathogens implicated in upper UTIs. The presence of acid-fast bacilli in a subset of patients highlights the importance of considering genitourinary tuberculosis as an underlying etiology in endemic regions. Antibiotic susceptibility patterns could not be systematically analyzed due to incomplete and non-uniform availability of culture sensitivity reports in this retrospective cohort, which represents a limitation of the study.

Urgent decompression remains the cornerstone of management in pyonephrosis. In routine clinical practice at our institution, most patients diagnosed with pyonephrosis undergo urinary decompression within 24-48 hours following initial resuscitation and optimization. However, precise time-to-decompression data could not be uniformly extracted from retrospective records, precluding formal analysis of this variable. PCN was the most frequently employed modality in our series and demonstrated high technical success. While both PCN and DJ stenting are effective in selected patients, evidence suggests that PCN is preferable in severely infected systems, marked hydronephrosis, heavy stone burden, or distorted anatomy. Studies by Zul Khairul Azwadi I et al. and subsequent meta-analyses have demonstrated comparable rates of sepsis resolution between PCN and DJ stenting, with higher technical success for PCN in complex obstruction [20]. Our institutional practice aligns with these findings and current guideline recommendations.

Renal salvage in pyonephrosis varies widely across studies, ranging from 20% to 60%, depending on the chronicity of obstruction and systemic involvement. The renal salvage rate of approximately 53% in our cohort is comparable with reports by Hemal AK et al. (~55%) [21].

Several clinical and imaging parameters were strongly associated with non-salvageability. Parenchymal thickness <5 mm was the strongest predictor of nephrectomy in our study, consistent with prior literature identifying cortical thickness as a surrogate marker of chronicity and residual renal function. Studies by Sharma U et al. and Bhat et al. have demonstrated that cortical thickness <5 mm correlates with irreversible damage and severely reduced split renal function on nuclear imaging [22]. Severe hydronephrosis was another significant predictor of non-salvageability, reflecting prolonged obstruction and ischemic injury, as previously demonstrated by Sharma U et al. [22]. Systemic factors such as sepsis and AKI at presentation were also strongly associated with nephrectomy, reinforcing the concept that a severely infected kidney with compromised parenchyma often functions as a source organ requiring removal to achieve definitive sepsis control, consistent with reports by Chen CC et al. and Kapoor R et al. [23,24]. Delayed presentation, reflected by longer symptom duration, significantly reduced the likelihood of renal recovery, consistent with reports by Kalra S et al. and Demirtaş A et al. [25,26].

Etiology also influenced outcomes. Stone-related pyonephrosis demonstrated significantly higher salvage rates, whereas xanthogranulomatous pyelonephritis, malignancy, and tubercular kidneys, conditions associated with extensive parenchymal destruction, showed a trend toward non-salvageability. These observations are in agreement with earlier reports by Malek RS et al., although statistical significance for some etiologies was limited by small subgroup sizes [27].

Western series often report lower nephrectomy rates, likely reflecting earlier access to diagnostic imaging and prompt intervention. In contrast, studies from the Indian subcontinent and other Asian regions consistently demonstrate higher nephrectomy rates, reflecting delayed diagnosis, limited access to healthcare, and advanced disease at presentation. Our findings fall within this spectrum and underscore the impact of socioeconomic and geographic factors on outcomes.

The strengths of the present study include a relatively large cohort from a high-volume tertiary referral center and a comprehensive evaluation of etiological factors, management strategies, and predictors of renal salvage.

This study has several limitations. Its retrospective design limits control over confounding variables and relies on the accuracy and completeness of medical records. As a single-centre experience from a tertiary referral hospital, the findings may be influenced by referral bias and may not be generalizable to other healthcare settings. The precise time from presentation to urinary decompression could not be systematically analyzed due to inconsistent documentation in emergency and referral records, despite most patients undergoing drainage after initial resuscitation. Antibiotic susceptibility patterns could not be uniformly assessed because culture sensitivity data were incomplete and non-standardized across the study period. Long-term functional renal outcomes were not evaluated owing to the lack of consistent post-discharge follow-up and serial renal function measurements. Finally, decisions regarding nephrectomy versus renal salvage were based on clinical, radiological, and intraoperative factors, which may have introduced selection bias, despite adherence to standardized institutional management protocols.

Overall, our findings emphasize the importance of early diagnosis, prompt decompression, preferably by PCN in high-risk patients, and timely definitive management to optimize renal salvage. Identification of simple clinical and imaging predictors may aid early risk stratification and guide decision-making, potentially improving outcomes in patients with pyonephrosis.

## Conclusions

In our tertiary-care experience, urolithiasis was the most common cause of pyonephrosis, with delayed presentation being frequent among rural patients. USG and CECT enabled early diagnosis, and prompt urinary decompression, most commonly via PCN in unstable or severely infected patients, was associated with effective sepsis control. Renal salvage was achieved in nearly half of the cases, demonstrating that not all pyonephrotic kidneys require nephrectomy. Acute kidney injury, marked parenchymal thinning, prolonged obstruction, and severe hydronephrosis were significantly associated with nephrectomy. These findings emphasize the importance of timely referral, early intervention, and definitive treatment of the underlying etiology to optimize renal outcomes.
